# Combination immunohistochemistry for SMAD4 and Runt-related transcription factor 3 may identify a favorable prognostic subgroup of pancreatic ductal adenocarcinomas

**DOI:** 10.18632/oncotarget.20815

**Published:** 2017-09-11

**Authors:** Yangkyu Lee, Hyejung Lee, Hyunjin Park, Jin-Won Kim, Jin-Hyeok Hwang, Jaihwan Kim, Yoo-Seok Yoon, Ho-Seong Han, Haeryoung Kim

**Affiliations:** ^1^ Department of Pathology, Seoul National University Bundang Hospital, Seoul National University College of Medicine, Seongnam, Republic of Korea; ^2^ Department of Pathology, Seoul National University Hospital, Seoul National University College of Medicine, Seoul, Republic of Korea; ^3^ Department of Internal Medicine, Seoul National University Bundang Hospital, Seoul National University College of Medicine, Seongnam, Republic of Korea; ^4^ Department of Surgery, Seoul National University Bundang Hospital, Seoul National University College of Medicine, Seongnam, Republic of Korea

**Keywords:** pancreatic ductal adenocarcinoma, SMAD4, RUNX3, immunohistochemistry, prognosis

## Abstract

**Purposes:**

SMAD4/DPC4 mutations have been associated with aggressive behavior in pancreatic ductal adenocarcinomas (PDAC), and it has recently been suggested that RUNX3 expression combined with SMAD4 status may predict the metastatic potential of PDACs. We evaluated the prognostic utility of SMAD4/RUNX3 status in human PDACs by immunohistochemistry.

**Materials and Methods:**

Immunohistochemical stains were performed for SMAD4 and RUNX3 on 210 surgically resected PDACs, and the results were correlated with the clinicopathological features.

**Results:**

Loss of SMAD4 expression was associated with poor overall survival (OS) (*p* = 0.015) and progression-free survival (PFS) (*p* = 0.044). Nuclear RUNX3 expression was associated with decreased OS (*p* = 0.010) and PFS (*p* = 0.009), and more frequent in poorly differentiated PDACs (*p* = 0.037). On combining RUNX3/SMAD4 status, RUNX3-/SMAD4+ PDACs demonstrated longer OS (*p* = 0.008, median time; RUNX3-/SMAD4+ 34 months, others 17 months) and PFS (*p* = 0.009, median time; RUNX3-/SMAD4+ 29 months, others 8 months) compared to RUNX3+/SMAD4+ and SMAD4- groups; RUNX3-/SMAD4+ was a significant independent predictive factor for both OS [*p* = 0.025, HR 1.842 (95% CI 1.079-3.143)] and PFS [*p* = 0.020, HR 1.850 (95% CI 1.100-3.113)].

**Conclusions:**

SMAD4-positivity with RUNX3-negativity was a significant independent predictive factor for favorable OS and PFS in PDAC. This is the first and large clinicopathological study of RUNX3/SMAD4 expression status in human PDAC. Combination immunohistochemistry for SMAD4 and RUNX3 may help identify a favorable prognostic subgroup of PDAC.

## INTRODUCTION

Pancreas cancer is the fourth most common cause of cancer-related deaths in industrialized countries, with an overall 5-year survival rate of about 8% [1]. The majority of patients are diagnosed at an advanced stage with distant metastasis or invasion of adjacent organs, and less than 20% of patients are amenable to curative resection [1].

The common genetic alterations in pancreatic carcinogenesis include activating mutations in *KRAS*, telomere shortening, and inactivation of *CDK2NA/p16*, *TP53* and *SMAD4* genes [2, 3]. However, the molecular landscape of pancreatic ductal adenocarcinomas (PDACs) is extremely vast. For example, recent large-scale genomic analyses on PDACs have identified various subtypes of these cancers highlighting their heterogeneity, e.g. the “Collisson class” (classical, quasimesenchymal and exocrine-like subtypes), “Moffitt tumor class” (basal-like versus classical), “Moffitt stromal class” (activated stroma, normal stroma) and the subtypes recently defined by Bailey et al. (squamous, pancreatic progenitor, immunogenic and aberrantly differentiated endocrine exocrine (ADEX) subtypes) [4–6]. Thus, targeting the specific molecular subtypes would ideally help improve the outcomes of PDAC patients, in the way that selected patients with breast and lung cancers have greatly benefitted from targeted therapy over the past decades.

While such high-throughput genomic analyses have yielded promising results, it is also important that inexpensive and easily applicable tests (e.g. immunohistochemical assays) are developed for predicting therapeutic response and prognosis. In this regard, Runt-related transcription factor 3 (RUNX3) is an interesting candidate biomarker for PDAC, as it has recently been demonstrated to play a role as a “metastatic switch” in PDACs in genetically engineered mouse models: RUNX3 was suggested to be an important determinant of the ability of PDAC cells to metastasize to distant sites or to proliferate locally [7, 8]. Moreover, RUNX3 expression was shown to be influenced by SMAD4 status in the same study: heterozygous deletion of *SMAD4* (+/−) attenuated RUNX3 levels compared to intact *SMAD4* (+/+) and homozygously-deleted *SMAD4* (−/−) [8].

If the combination of RUNX3 and SMAD4 status could predict the behavior of PDAC, analyzing RUNX3 and SMAD4 protein expression status by immunohistochemistry in resected or biopsied PDAC tissues would greatly benefit pancreatic cancer patients by helping to guide treatment plans. For example, PDACs with increased metastatic potential could benefit from systemic chemotherapy, while the more locally proliferative PDACs would be candidates for more aggressive local treatment, such as surgery or radiotherapy. Therefore, in this study, we performed an immunohistochemical analysis of RUNX3 and SMAD4 expression in our cohort of 210 surgically resected PDACs, and compared the clinicopathological features of PDACs according to RUNX3/SMAD4 expression status.

## RESULTS

### Clinicopathological characteristics

The clinicopathological characteristics of 210 cases are summarized in Table 1. 126 (60%) out of 210 patients were male, and the median age at initial operation was 65 years (range: 29~89 years). Most patients underwent operation for initial treatment. Only 7 patients received neoadjuvant chemotherapy before resection. The majority of PDACs were moderately differentiated (159/210, 75.7%) and were pT3 by the American Joint Committee on Cancer (AJCC) 7th edition (201/210, 95.7%). When the recently published size-based AJCC 8th edition was applied, 12.4% (26/210) were pT1 and 58.1% (122/210) were pT2, while 28.5% and 1.0% were pT3 and pT4, respectively, at the time of surgery. 130/210 (61.9%) cases had lymph node metastasis. On follow up, 86/210 (41.0%) and 121/210 (57.6%) developed local recurrence and distant metastasis, respectively, and 174/210 (82.9%) patients were deceased at last follow up.

**Table 1:** Patient and tumor characteristics (*n* = 210)

Sex	
Male	126 (60.0%)
Female	84 (40.0%)
Age at operation, median (range, years)	65 (29–89)
Initial CEA, median (ng/ml)	3.0 (0.01-50.6)
Initial CA19–9, median (IU/ml)	121.2 (0.01–10000)
Tumor size	
≤ 2 cm	26 (12.4%)
> 2 cm and ≤ 4 cm	123 (58.6%)
> 4 cm	61 (29.0%)
Preoperative treatment	
None	203 (96.7%)
Neoadjuvant chemotherapy	7 (3.3%)
Histologic grade	
Well differentiated	23 (11.0%)
Moderately differentiated	159 (75.7%)
Poorly differentiated	23 (11.0%)
Undifferentiated	5 (2.3%)
pT stage (AJCC 7th Ed.)	
pT1	2 (1.0%)
pT2	5 (2.3%)
pT3	201 (95.7%)
pT4	2 (1.0%)
pT stage (AJCC 8th Ed.)	
pT1 (≤ 2 cm)	26 (12.4%)
pT1a (≤ 0.5 cm)	0
pT1b (> 0.5 cm and <1 cm)	0
pT1c (≥ 1 cm and ≤ 2 cm)	26
pT2 (>2 cm and ≤ 4 cm)	122 (58.1%)
pT3 (>4 cm)	60 (28.5%)
pT4	2 (1.0%)
pN stage (AJCC 7th Ed.)	
pN0	80 (38.1%)
pN1	130 (61.9%)
pN stage (AJCC 8th Ed.)	
pN0 (no metastasis)	80 (38.1%)
pN1 (1~3)	97 (46.2%)
pN2 (≥ 4)	33 (15.7%)
Local recurrence	
Absent	124 (59.0%)
Present	86 (41.0%)
Distant metastasis	
Absent	89 (42.4%)
Present	121 (57.6%)
Status at last follow up	
Alive	36 (17.1%)
Deceased	174 (82.9%)

### RUNX3 and SMAD4 expression in pancreatic ductal adenocarcinomas

**Figure 1:**
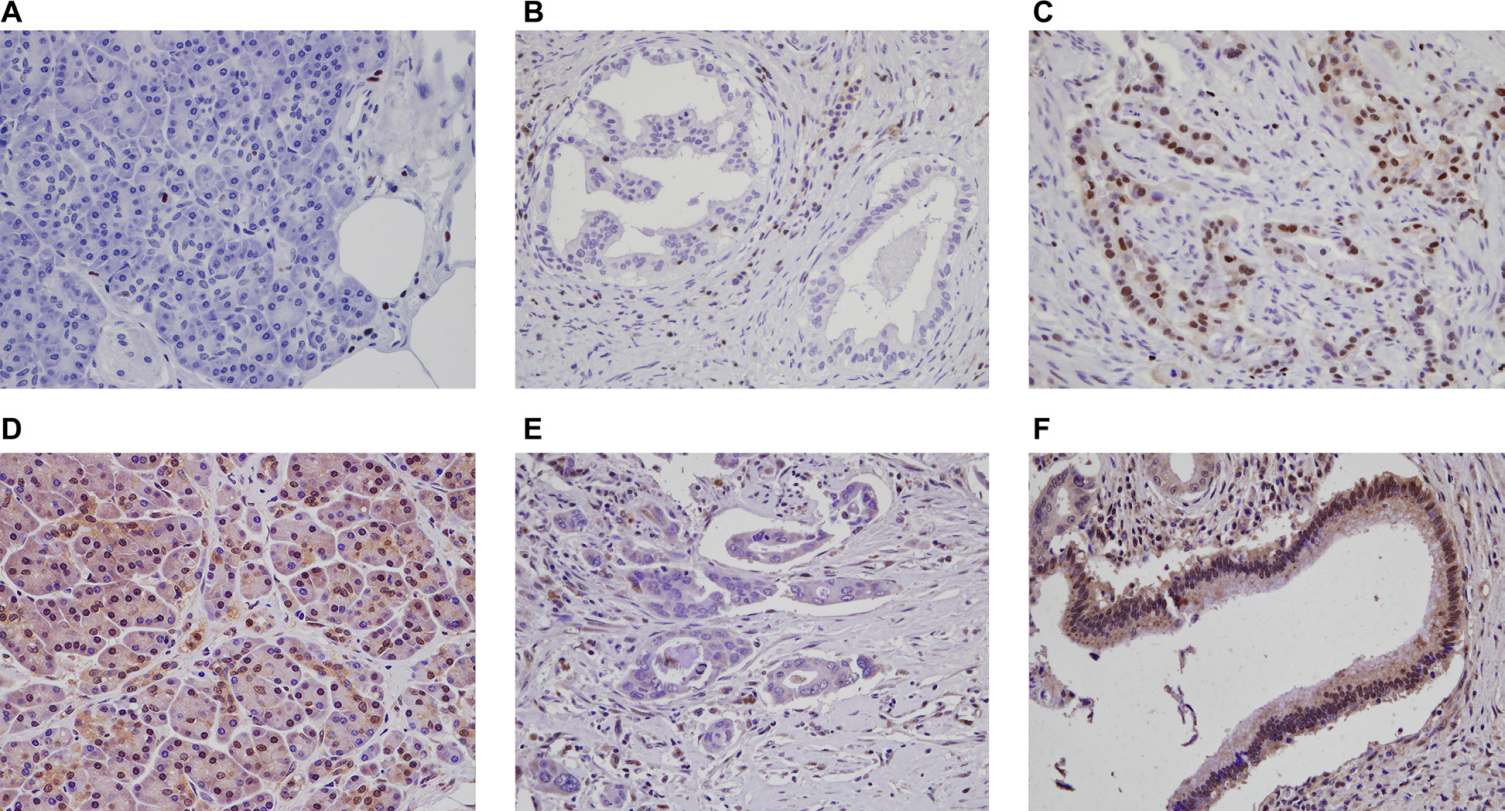
Immunohistochemical stain results for RUNX3 and SMAD4 in PDACs and normal pancreatic parenchyma (**A**) RUNX3 is not expressed in normal pancreatic tissue. A few scattered lymphocytes show nuclear RUNX3 expression. Representative PDACs with (**B**) no nuclear RUNX3 expression and (**C**) RUNX3 expression. (**D**) Intact SMAD4 expression in normal pancreatic tissue. PDACs with (**E**) SMAD4 loss, and (**F**) intact SMAD4 expression (original magnification x400).

The immunohistochemical stain results are demonstrated in Figure 1. Loss of SMAD4 expression (“SMAD4-”) was observed in 145 (72.5%) cases, while the remaining 55 (27.5%) cases had intact SMAD4 (“SMAD4+”). RUNX3 expression (“RUNX3+”) was identified in 121 cases (59.0%) compared with remaining 84 cases showing no RUNX3 expression (“RUNX3−”). On combining the SMAD4 and RUNX3 status, 25 (11.9%) PDACs demonstrated intact SMAD4 with no RUNX3 expression (RUNX3−/SMAD4+).

When the clinicopathological features were compared according to SMAD4 and RUNX3 expression status (Table 2), we found SMAD4 loss was associated with increased tumor size (*p* = 0.030), higher pN stage (AJCC 8th ed.) (*p* = 0.035), poor histological differentiation (*p* = 0.051) and higher pT stage (AJCC 8th ed.) (*p* = 0.054), although the latter two parameters were marginally significant. When the disease recurrence patterns were compared, PDACs with SMAD4 loss tended to result more frequently in distant metastasis compared to SMAD4-intact PDACs (*p* = 0.058), while there was no difference in the frequency of local recurrence according to SMAD4 expression status. RUNX3 expression was associated with tumor size (*p* = 0.039) and poor histological differentiation (*p* = 0.033).

**Table 2:** RUNX3 and SMAD4 status and clinicopathological features in pancreatic ductal adenocarcinoma cases

Parameters	SMAD4 loss (SMAD4-)	Intact SMAD4 (SMAD4+)	*p*-value	No RUNX3 expression (RUNX3-)	RUNX3 expression (RUNX3+)	*p*-value
Sex			0.747			0.774
Male	88 (60.7%)	32 (58.2%)		49 (58.3%)	73 (60.3%)	
Female	57 (39.3%)	23 (41.8%)		35 (41.7%)	48 (39.7%)	
Age at operation (mean ± SD)	65.1 ± 9.94	62.3 ± 9.92	0.082	65.3 ± 8.61	63.3 ± 10.96	0.146
Tumor size			**0.030**			**0.039**
≤ 2 cm	12 (8.3%)	12 (21.8%)		8 (9.5%)	17 (14.0%)	
> 2 cm and ≤ 4 cm	90 (62.0%)	28 (50.9%)		58 (69.0%)	62 (51.2%)	
> 4 cm	43 (29.7%)	15 (27.3%)		18 (21.4%)	42 (34.7%)	
Histologic grade			0.051			**0.033**
WD/MD	122 (84.1%)	52 (94.5%)		78 (92.9%)	100 (82.6%)	
PD/UD	23 (15.9%)	3 (5.5%)		6 (7.1%)	21 (17.4%)	
Lymphatic invasion			0.665			0.973
Absent	81 (56.3%)	29 (52.7%)		46 (54.8%)	66 (55.0%)	
Present	63 (43.7%)	26 (47.3%)		38 (45.2%)	54 (45.0%)	
Venous invasion			0.591			0.228
Absent	91 (63.2%)	37 (67.3%)		58 (69.0%)	73 (60.8%)	
Present	53 (36.8%)	18 (32.7%)		26 (31.0%)	47 (39.2%)	
Perineural invasion			0.850			0.926
Absent	22 (15.3%)	9 (16.4%)		13 (15.5%)	18 (15.0%)	
Present	122 (84.7%)	46 (83.6%)		71 (84.5%)	102 (85.0%)	
pT stage (AJCC 7th Ed.)			0.725			0.509
pT1	1 (0.7%)	0 (0.0%)		1 (1.2%)	0 (0.0%)	
pT2	4 (2.8%)	1 (1.8%)		3 (3.6%)	2 (1.7%)	
pT3	138 (95.2%)	54 (98.2%)		79 (94.0%)	118 (97.5%)	
pT4	2 (1.4%)	0 (0.0%)		1 (1.2%)	1 (0.8%)	
pT stage (AJCC 8th Ed.)			0.054			0.115
pT1	12 (8.3%)	12 (21.8%)		8 (9.5%)	17 (14.0%)	
pT2	89 (61.4%)	28 (50.9%)		57 (67.9%)	62 (51.2%)	
pT3	42 (29.0%)	15 (27.3%)		18 (21.4%)	41 (33.9%)	
pT4	2 (1.4%)	0 (0.0%)		1 (1.2%)	1 (0.8%)	
pN stage (AJCC 7th Ed.)			0.493			0.202
pN0	52 (36.6%)	23 (41.8%)		28 (33.3%)	51 (42.1%)	
pN1	93 (63.4%)	32 (58.2%)		56 (66.7%)	70 (57.9%)	
pN stage (AJCC 8th Ed.)			**0.035**			0.427
pN0	53 (36.6%)	23 (41.8%)		28 (33.3%)	51 (42.1%)	
pN1	73 (50.3%)	18 (32.7%)		42 (50.0%)	51 (42.1%)	
pN2	19 (13.1%)	14 (25.5%)		14 (16.7%)	19 (15.7%)	
Distant metastasis			0.058			0.427
Absent	55 (37.9%)	29 (52.7%)		38 (45.2%)	48 (39.7%)	
Present	90 (62.1%)	26 (47.3%)		46 (54.8%)	73 (60.3%)	
Local recurrence			0.364			0.682
Absent	82 (56.6%)	35 (63.6%)		51 (60.7%)	70 (57.9%)	
Present	63 (43.4%)	20 (36.4%)		33 (39.3%)	51 (42.1%)	

On combining the expression status of the two markers, PDACs with RUNX3-/SMAD4+ profiles showed less frequent distant metastasis (*p* = 0.017) and tended to be smaller in size (*p* = 0.092) compared to the other PDACs which showed SMAD4 loss and/or RUNX3 expression (Table 3).

**Table 3:** Relationship between RUNX3/SMAD4 combined status and clinicopathological features in pancreatic ductal adenocarcinoma cases

Parameters	RUNX3-/SMAD4+	Others* (RUNX3+SMAD4+, SMAD4- groups)	*p*-value
Sex			0.383
Male	13 (52.0%)	107 (61.1%)	
Female	12 (48.0%)	68 (38.9%)	
Age at operation (mean ± SD)	65.2 ± 8.36	64.2 ± 10.22	0.605
Tumor size			0.092
≤ 2 cm	5 (20.0%)	19 (10.9%)	
> 2 cm and ≤ 4 cm	17 (68.0%)	101 (57.7%)	
> 4 cm	3 (12.0%)	55 (31.4%)	
Histologic grade			0.153
WD/MD	24 (96.0%)	150 (85.7%)	
PD/UD	1 (4.0%)	25 (14.3%)	
Lymphatic invasion			0.611
Absent	15 (60.0%)	95 (54.6%)	
Present	10 (40.0%)	79 (45.4%)	
Venous invasion			0.681
Absent	17 (68.0%)	111 (63.8%)	
Present	8 (32.0%)	63 (36.2%)	
Perineural invasion			1.000
Absent	4 (16.0%)	27 (15.5%)	
Present	21 (84.0%)	147 (84.5%)	
pT stage (AJCC 7th Ed.)			0.755
pT1	0 (0.0%)	1 (0.6%)	
pT2	0 (0.0%)	5 (2.9%)	
pT3	25 (100.0%)	167 (95.4%)	
pT4	0 (0.0%)	2 (1.1%)	
pT stage (AJCC 8th Ed.)			0.173
pT1	5 (20.0%)	19 (10.9%)	
pT2	17 (68.0%)	100 (57.1%)	
pT3	3 (12.0%)	54 (30.9%)	
pT4	0 (0.0%)	2 (1.1%)	
pN stage (AJCC 7th Ed.)			0.509
pN0	11 (44.0%)	65 (37.1%)	
pN1	14 (56.0%)	110 (62.9%)	
pN stage (AJCC 8th Ed.)			0.592
pN0	11 (44.0%)	65 (37.1%)	
pN1	9 (36.0%)	82 (46.9%)	
pN2	5 (20.0%)	28 (16.0%)	
Distant metastasis			**0.017**
Absent	16 (64.0%)	68 (38.9%)	
Present	9 (36.0%)	107 (61.1%)	
Local recurrence			0.303
Absent	17 (68.0%)	100 (57.1%)	
Present	8 (32.0%)	75 (42.9%)	

*Others: RUNX3-/SMAD4-, RUNX3+/SMAD4−, RUNX3+/SMAD4+.

### Survival analysis

**Figure 2:**
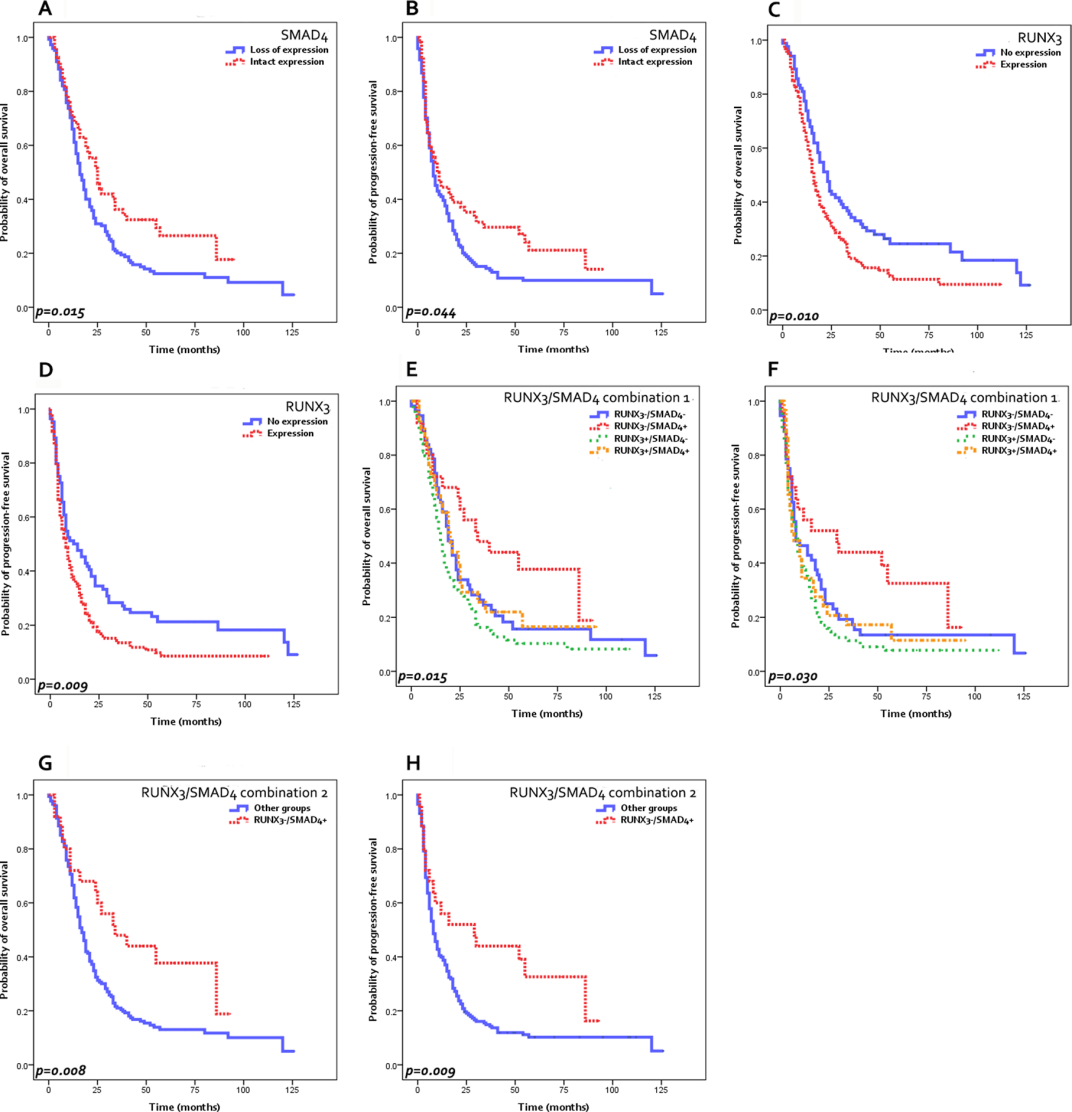
Survival analysis results Decreased overall survival (OS) (**A**) and progression-free survival (PFS) (**B**) are seen in PDACs with SMAD4 loss compared to those with intact SMAD4. RUNX3-expressing PDACs demonstrate decreased OS (**C**) and PFS (**D**) compared to RUNX3-negative tumors. RUNX3−/SMAD4+ PDACs show increased OS (**E**, **G**) and PFS (**F**, **H**) compared to the other PDACs. (**G**, **H**) “Other groups”: RUNX3−/SMAD4−, RUNX3+/SMAD4−, RUNX3+/SMAD4+ PDACs).

The univariate survival analysis results are summarized in Table 4 and Figure 2. Clinicopathological parameters significantly associated with poor overall survival included large tumor size (*p* = 0.032), presence of lymphatic (*p* = 0.005), venous (*p* < 0.001) or perineural invasion (*p* = 0.007), high pT stage (7th ed.) (*p* = 0.041) and high pN stage (7th ed. *p* < 0.001, 8th ed. *p* = 0.001). Similar findings were seen for progression-free survival: larger tumor size (*p* = 0.013), presence of lymphatic (*p* = 0.003), venous (*p* = 0.003) or perineural invasion (*p* = 0.007), high pT stage (7th ed. *p* = 0.014, 8th ed. *p* = 0.018), and high pN stage (7th ed. *p* = 0.003, 8th ed. *p* = 0.011) were significantly associated with reduced progression-free survival.

**Table 4:** Survival analysis in clinicopathological variables and immunohistochemical results

Parameters	Overall survival			Progression–free survival		
	Median survival time (month, 95% CI)	Number of events	*p*-value	Median survival time (month, 95% CI)	Number of events	*p*-value
Sex			0.169			0.189
Male	19.00 (15.81–22.18)	111/126		10.00 (6.10–13.89)	113/125	
Female	17.00 (11.40–22.59)	63/84		8.00 (6.36–9.63)	67/84	
Age at operation			0.376			0.715
≤ 65 years	19.00 (13.96–24.03)	88/108		10.00 (6.40–13.59)	91/107	
< 65 years	16.00 (12.27–19.72)	86/102		8.00 (5.67–10.32)	89/102	
Tumor size			**0.032**			**0.013**
≤ 2cm	29.00 (19.00–38.99)	20/26		16.00 (9.82–22.17)	21/26	
> 2cm and ≤ 4cm	19.00 (15.40–22.59)	103/123		10.00 (5.67–14.32)	105/122	
> 4cm	12.00 (8.79–15.20)	51/61		5.00 (3.49–6.50)	54/61	
Histologic grade			0.124			0.391
WD/MD	19.00 (15.59–22.40)	150/182		9.00 (6.89–11.10)	157/182	
PD/UD	11.00 (7.12–14.87)	24/28		5.00 (1.60–8.39)	23/27	
Lymphatic invasion			**0.005**			**0.003**
Absent	21.00 (16.51–25.48)	91/114		15.00 (10.83–19.16)	94/113	
Present	14.00 (11.44–16.44)	83/95		6.00 (3.96–8.03)	86/95	
Venous invasion			**< 0.001**			**0.003**
Absent	23.00 (19.05–26.94)	106/134		11.00 (6.83–15.16)	111/133	
Present	13.00 (10.52–15.47)	68/75		6.00 (4.14–7.86)	69/75	
Perineural invasion			**0.007**			**0.007**
Absent	24.00 (0.00–59.25)	20/32		19.00 (2.36–35.63)	22/32	
Present	17.00 (14.66–19.34)	154/177		8.00 (6.00–9.99)	158/176	
pT stage (7th Ed.)			**0.041**			**0.014**
pT1	8.00	1/2		8.00	1/2	
pT2	*	1/5		*	1/5	
pT3	18.00 (15.62–20.37)	170/201		9.00 (7.09–10.90)	176/200	
pT4	14.00	2/2		*	2/2	
pT stage (8th Ed.)			0.055			**0.018**
pT1	29.00 (19.00–38.99)	20/26		16.00 (9.82–22.17)	21/26	
pT2	19.00 (15.42–22.57)	102/122		10.00 (5.69–14.30)	104/121	
pT3	12.00 (8.82–15.17)	50/60		5.00 (3.39–6.60)	53/60	
pT4	14.00	2/2		4.00	2/2	
pN stage (7th Ed.)			**< 0.001**			**0.003**
pN0	27.00 (16.02–37.97)	59/80		14.00 (7.22–20.77)	63/79	
pN1	16.00 (13.64–18.35)	115/130		8.00 (6.04–9.95)	117/130	
pN stage (8th Ed.)			**0.001**			**0.011**
pN0	27.00 (16.02–37.97)	59/80		14.00 (7.22–20.77)	63/79	
pN1	15.00 (12.62–17.37)	85/97		8.00 (6.41–9.58)	87/97	
pN2	17.00 (13.78–20.21)	30/33		9.00 (4.98–13.01)	30/33	
SMAD4 status			**0.015**			**0.044**
Loss	16.00 (13.54–18.45)	127/145		8.00 (5.95–10.04)	130/144	
Intact	25.00 (19.15–30.84)	39/55		11.00 (6.21–15.78)	42/55	
RUNX3 status			**0.010**			**0.009**
No expression	23.00 (18.51–27.48)	66/84		12.00 (5.41–18.58)	68/84	
Expression	16.00 (13.57–18.42)	104/121		8.00 (5.53–10.46)	108/120	
4–group combination			**0.015**			**0.030**
RUNX3–/SMAD4–	19.00 (14.92–23.07)	48/56		8.00 (0.67–15.32)	49/56	
RUNX3–/SMAD4+	34.00 (12.78–55.21)	16/25		29.00 (0.00–58.37)	17/25	
RUNX3+/SMAD4–	15.00 (12.74–17.25)	79/89		8.00 (5.70–10.29)	81/88	
RUNX3+/SMAD4+	20.00 (13.72–26.27)	23/30		7.00 (1.72–12.27)	25/30	
2–group combination			**0.008**			**0.009**
RUNX3–/SMAD4+	34.00 (12.78–55.21)	16/25		29.00 (0.00–58.37)	17/25	
Others**	17.00 (14.72–19.27)	150/175		8.00 (6.01–9.98)	155/174	

*All cases were censored.

** RUNX3–/SMAD4–, RUNX3+/SMAD4–, RUNX3+/SMAD4+.

When the survival was analyzed according to the immunohistochemical stain results, loss of SMAD4 expression was significantly associated with poor overall survival (median survival 16 months versus 25 months, *p* = 0.015) and progression-free survival (median survival 8 months versus 11 months, *p* = 0.044) compared to PDACs with intact SMAD4 expression. RUNX3 expression was significantly associated with decreased overall survival (median survival 16 months versus 23 months, *p* = 0.010) and progression-free survival (median survival 8 months versus 12 months, *p* = 0.009) compared to RUNX3-negative PDACs. When the RUNX3/SMAD4 status was combined, RUNX3-/SMAD4+ tumors were associated with remarkably longer overall survival (median 34 months versus 17 months, *p* = 0.008) and progression-free survivals (median 29 months versus 8 months, *p* = 0.009) compared to other groups. Interestingly, among the PDACs with no RUNX3 expression, those with SMAD4 loss demonstrated similarly decreased overall and progression-free survivals as RUNX3-expressing PDACs.

After adjusting for sex and age, RUNX3-/SMAD4+ status remained a significant independent predictive factor for favorable overall [*p* = 0.025, HR 1.842 (95% CI 1.079–3.143)] and progression-free survival [*p* = 0.020, HR 1.850 (95% CI 1.100–3.113)] on multivariate analysis, in addition to tumor size (Table 5).

**Table 5:** Multivariate analysis for overall survival and progression-free survival

Parameters	Overall survival		Progression-free survival	
	Hazard ratio (95% CI)	*p*-value	Hazard ratio (95% CI)	*p*-value
Variables adjusted for age and sex				
RUNX3−/SMAD4+ vs. others*	1.842 (1.079–3.143)	**0.025**	1.850 (1.100–3.113)	**0.020**
Histologic grade	1.517 (0.961–2.393)	0.073	1.163 (0.730–1.852)	0.525
Tumor size				
≤ 2 cm	1	Reference	1	Reference
> 2 cm and ≤ 4 cm	1.511 (0.912–2.503)	0.109	1.298 (0.787–2.142)	0.307
> 4 cm	2.251 (1.287–3.935)	**0.004**	2.191 (1.248–3.847)	**0.006**
Lymphatic invasion	1.330 (0.962–1.839)	0.085	1.492 (1.101–2.023)	**0.010**
Venous invasion	1.547 (1.100–2.176)	**0.012**	1.267 (0.896–1.792)	0.180
Perineural invasion	1.546 (0.935–2.557)	0.089	1.565 (0.982–2.494)	0.059
pT stage (7th Ed.)	8.008 (1.071–59.866)	0.043	10.211 (1.380–75.567)	**0.023**
pN stage (7th Ed.)	1.178 (0.837–1.658)	0.348	1.057 (0.758–1.474)	0.745

*RUNX3-/SMAD4-, RUNX3+/SMAD4-, RUNX3+/SMAD4+.

### Comparison of immunohistochemical status between primary and metastatic lesions

Of the 210 patients studied, 121 (57.6%) subsequently developed distant metastases. Biopsies or resected tissues from metastatic sites were available for 14/121 patients and the whole tissue sections of the 14 metastatic tumors were subjected to SMAD4 and RUNX3 immunohistochemistry. The liver was the most common site of distant metastasis (*n* = 9) (Table 6).

**Table 6:** Comparison of SMAD4 and RUNX3 labeling status between primary and metastatic tumors (*n* = 14)

Case#	Status of primary site	Metastatic sites	Status of metastatic site
1	RUNX3+/SMAD4−	Liver	RUNX3−/SMAD4−
2	RUNX3+/SMAD4−	Liver	RUNX3−/SMAD4−
**3**	**RUNX3+/SMAD4**−	**Liver**	**RUNX3+/SMAD4−**
4	RUNX3+/SMAD4−	Cervical lymph node	RUNX3−/SMAD4−
5	RUNX3+/SMAD4−	Lung	RUNX3−/SMAD4−
6	RUNX3+/SMAD4−	Liver	RUNX3+/SMAD4+
7	RUNX3+/SMAD4−	Liver	RUNX3−/SMAD4−
**8**	**RUNX3-/SMAD4**−	**Liver**	**RUNX3−/SMAD4−**
9	RUNX3+/SMAD4+	Liver	RUNX3−/SMAD4−
**10**	**RUNX3-/SMAD4**−	**Liver**	**RUNX3−/SMAD4−**
**11**	**RUNX3-/SMAD4**−	**Liver**	**RUNX3−/SMAD4−**
12	RUNX3+/SMAD4+	Bone	RUNX3−/SMAD4−
13	RUNX3+/SMAD4−	Lung	RUNX3−/SMAD4−
**14**	**RUNX3-/SMAD4**−	**Colon**	**RUNX3−/SMAD4−**

Identical RUNX3/SMAD4 profiles were seen in the primary and metastatic tumors of 5/14 (35.7%) cases. While 11/14 (78.6%) of the cases showed concordant SMAD4 expression status between the matched primary and metastatic tumors, only 6/14 (42.9%) cases showed concordant RUNX3 expression status. Interestingly, out of the 14 matched cases, the RUNX3-/SMAD4+ profile was seen in none of the primary PDACs and also in none of their subsequent metastatic tumors. In addition, only 1/14 (7.1%) of the metastatic tumors had intact SMAD4 expression, and all 8 (100%) cases with discordant RUNX3 status were RUNX3+ in the primary tumor and RUNX3-negative in the matched metastatic tumors.

## DISCUSSION

The purpose of this study was to evaluate the expression status of SMAD4 and RUNX3 in PDACs, and to explore the utility of the SMAD4/RUNX3 marker combination assay in predicting PDAC behavior. We demonstrate in this retrospective study of 210 PDACs that the combination of intact SMAD4 and RUNX3 negativity may help to identify a subset of PDAC patients with favorable prognosis.

*SMAD4* (or *DPC4*) is a well-known tumor suppressor gene that is inactivated in the late stages of pancreatic carcinogenesis: *SMAD4* inactivation has been reported in ~50% of PDACs and in ~30% of high-grade pancreatic intraepithelial neoplasia (PanIN-3) [9–12]. Although the majority of clinicopathological studies, including our present study, have demonstrated associations between SMAD4 loss in PDACs and poor prognoses, there are also reports showing no significant correlations with survival, or even evidence for improved survival in PDACs with SMAD4 loss [13–17]. Loss of SMAD4 protein expression has been demonstrated to be a sensitive and specific surrogate marker for *SMAD4* alteration, independent of whether *SMAD4* inactivation occurred by homozygous deletions or by loss of heterozygosity, and the presence of intact SMAD4 protein expression corresponds to tumors with at least one wild-type allele (i.e. *SMAD4(+/+)* or *SMAD4(+/*−)) [11, 18]. Therefore, PDACs with intact SMAD4 protein expression would comprise a mixed population of *SMAD4(+/+)* and *SMAD4(+/*−) PDACs, and hence the discrepancies in the clinicopathological studies on the prognostic significance of SMAD4 immunohistochemistry are not surprising. In addition, PDACs with SMAD4 loss tended to show more frequent distant metastasis on follow-up compared to SMAD4-intact tumors, while there was no difference in the frequency of local progression according to SMAD4 status, supporting the previous literature that suggested correlations between SMAD4 status and disease progression patterns in PDAC [19, 20].

The role of RUNX3 in cancer is still not well-established; it has been described to function as a tumor suppressor in most malignancies, including cancers of the breast, stomach, liver, lung and prostate, [21–25] but tumor promoting roles of RUNX3 have been suggested for some tumors such as ovarian cancers, melanomas and PDACs [8, 26–28]. An association between RUNX3 protein expression and decreased overall survival has also been demonstrated in PDAC patients, although in a small number of cases [8]. A recent genetically engineered mouse model study by Whittle et al. demonstrated an interesting relationship between SMAD4 and RUNX3 status and the biological behavior (metastasis versus primary tumor growth) of PDACs according to SMAD4/RUNX3 status [7, 8, 29]. In brief, *KPC* (*SMAD4(+/+)*) and *KPDDC* (*SMAD4(*−*/*−)) tumors showed increased *RUNX3* expression levels and a high metastatic disease burden, and the high expression of *RUNX3* was associated with increased metastatic potential of PDACs independent of epithelial-mesenchymal transition [8, 30]. On the other hand, SMAD4 haploinsufficient *KPDC* mice (*SMAD4(+/*−)) lacked RUNX3 expression, had a significantly lower metastatic burden, but showed an increased proliferative activity of the primary tumor compared to *KPC* tumors. We did not find differences in the proliferative activity (Ki-67 labeling index) of PDACs according to SMAD4/RUNX3 status in this study (data not shown), but we did find that PDACs with RUNX3 expression and/or SMAD4 loss were more likely to metastasize to distant sites and follow an aggressive clinical course compared to PDACs that did not show these immunophenotypes. Therefore, combining the SMAD4 and RUNX3 protein expression status would provide a more accurate prognostic marker compared to SMAD4 status alone.

It is also interesting that when the immunohistochemical profiles of the primary and metastatic tumors were compared in the separate analysis of 14 primary versus matched metastatic PDACs, SMAD4 labeling status was concordant in the majority of the cases, while the RUNX3 expression status were more frequently discordant. All primary tumors showed RUNX3 expression and/or SMAD4 loss; RUNX3-/SMAD4+ status was not seen in any of the primary or metastatic tumors in this analysis. In addition, all RUNX3-discordant cases showed conversion from RUNX3-positivity in the primary tumor to RUNX3-negativity in the matched metastatic tumors. Attenuation of *RUNX3* levels once the metastatic niche is established, switching the metastatic tumor to the locally proliferative state, would be an attractive explanation for this observation; however, experimental evidence would be required to support this.

This is to our knowledge the first and the largest single institutional clinicopathological study demonstrating RUNX3/SMAD4 combination immunohistochemistry to be a useful predictor of both overall and disease-free survivals in PDAC patients. Although further validation in independent cohorts would be required, application of RUNX3/SMAD4 immunohistochemistry on biopsies or resected PDAC tissues may not only be a prognostic marker but also help guide treatment plans in PDAC patients; RUNX3−/SMAD4+ PDACs are less likely to metastasize to distant sites and may therefore be candidates for more aggressive local surgery and radiotherapy, whereas PDACs with RUNX3 expression and/or SMAD4 loss profiles may benefit more from systemic chemotherapy.

## MATERIALS AND METHODS

### Patient selection and clinicopathological analysis

This retrospective analysis was approved by the Institutional Review Board of Seoul National University Bundang Hospital (IRB #B-1611-369-302). The study subjects comprised 210 consecutive cases of PDACs resected between June 2003 and January 2013 at Seoul National University Bundang Hospital, Seongnam, South Korea. Clinicopathological data were retrieved by reviewing electronic medical records, pathology reports and glass slides, and included patient sex, initial treatment, age at operation, tumor size, histologic differentiation, serum tumor marker levels (CEA, CA19–9), and pathological T and N stages according to American Joint Committee on Cancer (AJCC) 7th edition and the new 8th edition. Follow up data was also retrieved from the electronic medical records, including the status at last follow up, and occurrence of local recurrence and/or distant metastasis during the follow up period. The dates of local recurrence and distant metastasis were defined as when new lesions appeared on imaging studies or when tumor marker levels were elevated. The date of disease progression was defined as when the event has occurred postoperatively such as local recurrence, distant metastasis or death.

### Immunohistochemistry

Two mm-core tissue microarrays were constructed from 210 PDACs and their matched non-neoplastic pancreatic tissues (Superbiochips Laboratories, Seoul, Korea), and 4 μm-thick tissue sections obtained from the microarray blocks were subjected to immunohistochemical staining for SMAD4 (1:100, rabbit monoclonal, Abcam, Cambridge, UK) and RUNX3 (1:100, rabbit monoclonal, Cell signaling technology, Denver, USA). In brief, tissue sections were deparaffinized in xylene, rehydrated in graded alcohol, and antigen retrieval was performed using citrate buffer (pH 6.0). Incubation with primary antibodies was performed for 1 hour, and then with secondary antibodies (EnVision Detection System, Dako) for 30 minutes. Counterstaining was performed using Mayer's hematoxylin.

The labeled slides were interpreted by 2 pathologists (YL and HK). SMAD4 was expressed in the cytoplasm and nuclei of normal pancreatic duct epithelia, acinar cells, lymphocytes, stromal fibroblasts and endocrine cells, and served as positive controls. Expression in < 20% of tumor cell nuclei was defined as SMAD4 loss (“SMAD4-”). RUNX3 was not expressed in normal pancreatic ductal epithelial cells or acinar cells, but was expressed in the lymphocytes. RUNX3 expression status was evaluated in terms of both intensity and distribution of staining. The staining intensity was defined as follows: “0”, no expression; “1”, weaker intensity compared to staining of lymphocytes; “2”, strong intensity similar to that seen in lymphocytes. For distribution, the percentage of positively stained tumor cell nuclei was estimated in 5% increments. The staining distribution was relatively homogeneous throughout the tumor. The presence of weak or strong RUNX3 positivity in 5% or more of tumor cells were regarded as positive (“RUNX3+”) for subsequent analysis.

For cases where both primary pancreatic tumors and metastatic tumors were available for examination, whole tissue sections were subjected to the same immunohistochemical stains for RUNX3 and SMAD4, and the expression status of both markers were compared between the primary and matched metastatic tumors. These cases were from the same cohort of 210 PDACs.

### Statistical analysis

All statistical analyses were performed using SPSS 19.0K (SPSS Korea, Seoul, South Korea). Chi-square tests, Fisher exact tests and Student *t*-tests were performed as deemed appropriate. Univariate survival analyses for overall and progression-free survivals were performed by the Kaplan-Meier method and log-rank test. The Cox regression models were used for multivariate analysis. Statistical significance was defined as *p* < 0.05.
